# The Identification of Stemness-Related Genes in the Risk of Head and Neck Squamous Cell Carcinoma

**DOI:** 10.3389/fonc.2021.688545

**Published:** 2021-06-11

**Authors:** Guanying Feng, Feifei Xue, Yingzheng He, Tianxiao Wang, Hua Yuan

**Affiliations:** ^1^ Jiangsu Key Laboratory of Oral Diseases, Nanjing Medical University, Nanjing, China; ^2^ Department of Oral and Maxillofacial Surgery, Affiliated Hospital of Stomatology, Nanjing Medical University, Nanjing, China

**Keywords:** head and neck squamous cell carcinoma, cancer stemness, risk, machine learning, compounds

## Abstract

**Objectives:**

This study aimed to identify genes regulating cancer stemness of head and neck squamous cell carcinoma (HNSCC) and evaluate the ability of these genes to predict clinical outcomes.

**Materials and Methods:**

The stemness index (mRNAsi) was obtained using a one-class logistic regression machine learning algorithm based on sequencing data of HNSCC patients. Stemness-related genes were identified by weighted gene co-expression network analysis and least absolute shrinkage and selection operator analysis (LASSO). The coefficient of LASSO was applied to construct a diagnostic risk score model. The Cancer Genome Atlas database, the Gene Expression Omnibus database, Oncomine database and the Human Protein Atlas database were used to validate the expression of key genes. Interaction network analysis was performed using String database and DisNor database. The Connectivity Map database was used to screen potential compounds. The expressions of stemness-related genes were validated using quantitative real‐time polymerase chain reaction (qRT‐PCR).

**Results:**

TTK, KIF14, KIF18A and DLGAP5 were identified. Stemness-related genes were upregulated in HNSCC samples. The risk score model had a significant predictive ability. CDK inhibitor was the top hit of potential compounds.

**Conclusion:**

Stemness-related gene expression profiles may be a potential biomarker for HNSCC.

## Introduction

Head and neck squamous cell carcinoma (HNSCC) was one of the most common cancers worldwide, with ~870,000 new cases and ~440,000 deaths in 2020 (1). It involves cancers of the oral cavity (40%), pharynx (25%), and larynx (15%). The major risk factors for HNSCC include betel nut chewing, alcohol consumption, tobacco consumption, and HPV infection (2).

Cancer stemness (CS) postulates that the growth of a tumor is fueled by limited numbers of dedicated stem cells capable of self-renewal. It represents the degree of oncogenic dedifferentiation (3, 4). Many studies have revealed the molecular mechanisms of CS. Redox and JAK/STAT3 signaling pathways were found to influence breast cancer stem cell state (5, 6). The Osteopontin-CD44 signaling pathway was found to enhance cancer stem cell phenotypes in glioma (7). Prostaglandin E2 was found to promote colorectal cancer stem cell expansion (8). Epithelial–mesenchymal transition (EMT) induced mitochondrial fusion through regulation of the miR200c-PGC1α-MFN1 pathway, directing the asymmetric division of mammary stem cells (9). Previous studies have shown that Oct4, Sox2 and Nanog were associated with oral cancer stem-like status (10, 11). Increasing evidence points to the significance of stemness-related biomarkers in solid tumors, but their role in the risk of HNSCC has not been evaluated. It is essential to identify stemness-related biomarkers and underlying mechanisms associated with CS of HNSCC.

A one-class logistic regression (OCLR) machine learning algorithm published recently was a useful method to quantify cancer stemness index. In this algorithm, a predictive model was constructed and trained in stem cell samples, which can be applied to tumor samples (4). Previous studies have shown the application of the OCLR algorithm in some cancers (12–14). Weighted gene co-expression network analysis (WGCNA) identifies gene modules related to clinical traits through clustering thousands of genes, then uses intramodular connectivity and gene significance based on the correlation of a gene expression profile with a sample trait to identify stemness-related genes for further research. Hence WGCNA identifies significant genes in the biological process more efficiently (15). The least absolute shrinkage and selection operator (LASSO) is a regression analysis method that performs both variable selection and regularization. It enhances the prediction accuracy and interpretability of the resulting statistical model (16, 17).

**Figure 1:**
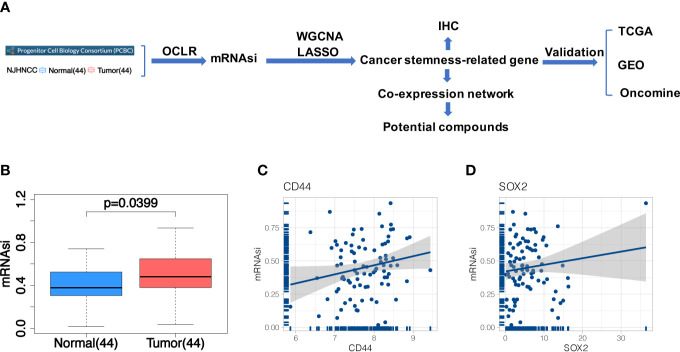
**(A)** The flowchart of the study; **(B)** Differences of mRNAsi between 44 tumor and 44 matched normal samples from NJHNCC. Line in the box indicates the median and boxes correspond with the first and third quartiles; **(C, D)** Correlation analysis between mRNAsi and HNSCC stemness marker (CD44, SOX2). It indicated the good fitness of mRNAsi with HNSCC stemness.

In this study, a systematic analysis of the relationship between the stemness-related genes and the risk of HNSCC was provided. A flow chart outlining the whole study is shown in **Figure 1A**.

## Materials and Methods

### Study Subjects

Some 44 newly diagnosed head and neck squamous cell carcinoma (HNSCC) patients without any treatment before the operation were enrolled from the Stomatology Hospital of Nanjing Medical University (Nanjing head and neck cancer cohort, NJHNCC). Surgically resected tumors and adjacent normal oral epithelial tissues were obtained from these patients. Clinical information was obtained through medical records while demographic information including age, gender, smoking, alcohol and Betel nut chewing history was collected from each patient using a standard questionnaire. Frozen tumors and matched normal specimens were confirmed by two independent pathologists. Samples were frozen in liquid nitrogen. HNSCC tissues with the malignant cell purity of over 70% were selected for RNA extraction and sequencing. The study was approved by the institutional review board of Nanjing Medical University. Clinical and demographic information is provided in **Table 1**.

**Table 1:** Demographic characteristics of 44 HNSCC patients from NJHNCC.

Characteristics	NJHNCC (n = 44)
Age (median; range)	48.5 (27–69)
Gender (frequency; percentage)	
Male	44 (100)
Female	0 (0)
Smoking history (frequency; percentage)	
Ever	40 (91)
Never	4 (9)
Alcohol (frequency; percentage)	
Ever	30 (68)
Never	14 (32)
Betel nut chewing history (frequency; percentage)	
Ever	39 (89)
Never	5 (11)
Clinical stage (frequency; percentage)	
I	0 (0)
II	6 (13.6)
III	21 (47.7)
IV	7 (15.9)
V	8 (18.2)
N/A	2 (4.5)

### Total RNA Extraction and RNA Sequencing

Total RNA was extracted from 44 matched tumor-normal samples using the RNeasy Mini Kit (Qiagen, Hilden, Germany). The quality and quantity of extracted RNA were assessed using the NanoDrop 2000 (Thermo Fisher Scientific, Wilmington, DE, USA), Qubit 2.0 Fluorometer (Life Technologies, CA, USA) and 1% agarose gel electrophoresis. RNA integrity was assessed using the RNA Nano 6000 Assay Kit (Agilent Technologies, CA, USA).

A total of 3 μg of high-quality RNA per sample was used for ribosomal RNA removal by the Epicentre Ribo-zero rRNA Removal Kit (Epicentre, USA) and the sequencing library was prepared using the rRNA-depleted RNA by the NEBNext Ultra Directional RNA Library Prep Kit for Illumina (NEB, USA) following manufacturer’s recommendations. The clustering of the index-coded samples was performed on a cBot Cluster Generation System using TruSeq PE Cluster Kit v3-cBot-HS (Illumina, San Diego, CA, USA), followed by the 150-bp paired-end sequencing on the HiSeq X Ten instrument (Illumina, San Diego, CA, USA) according to the manufacturer’s protocols. The result was reported in FPKM.

### Data Collection

RNA-seq data from the Progenitor Cell Biology Consortium (PCBC) database (https://www.synapse.org/#!Synapse:syn2701943) was obtained (18, 19). RNA-seq data of 500 HNSCC patients was obtained from The Cancer Genome Atlas (TCGA) database (https://portal.gdc.cancer.gov). The gene expression profiles of 167 HNSCC patients (GSE30784) were obtained from the Gene Expression Omnibus (GEO) database (https://www.ncbi.nlm.nih.gov/geo/) (20).

### Deriving Stemness Index (mRNAsi) Using Machine Learning

Briefly, the original algorithm was obtained from the previously published study (4). We derived stemness signatures using the one-class logistic regression (OCLR) machine learning algorithm trained on gene expression profiles of stem cell samples from the PCBC database. We applied the stemness signatures to RNA-seq data of NJHNCC and obtained stemness index (mRNAsi). The higher mRNAsi, the higher degree of stemness.

The workflow to generate stemness index was as follows: a) download gene expression profiles of stem cell samples from PCBC database (syn2701943); b) map Ensembl IDs to HUGO; c) identify stem cell sample; d) train a one-class logistic regression model; e) perform leave-one-out cross-validation of the one-class logistic regression model; f) export stemness signatures; g) load and preprocess NJHNCC-RNAseq data; h) calculate raw stemness index using Spearman’s correlation; i) standardization of raw stemness index; and j) export stemness index in the interval (0,1). The process was done in R 3.6.1.

The flowchart of deriving stemness index (mRNAsi) was shown in **Supplemental Figure 1**. The R code was shown in **Supplemental File R code**. The stemness signatures of the predictive model were shown in **Supplemental Table 1**.

Wilcoxon test was used to determine the significance of mRNAsi differences between tumor and normal samples. The representative stemness markers of HNSCC were obtained from a published study (21). The correlations between mRNAsi and HNSCC stemness markers were calculated based on the Linear regression (21).

### Differential Gene Expression Analysis

The R limma package was used to screen differentially expressed genes between normal and tumor samples. The Wilcoxon signed-rank test was used to identify the differentially expressed genes. The RNA-seq data was transformed by log_2_ (x + 1) for normalization. The screening criteria was |log_2_ fold change (FC)| >1 and the false discovery rate (FDR) <0.05.

### Weighted Gene Co-Expression Network Analysis

WGCNA was conducted with differentially expressed genes. Differentially expressed gene profiles were screened to delete outliers. A correlation matrix was constructed with the absolute values in transcriptome data. An adjacency matrix was constructed by the soft threshold. A topological overlap matrix was then constructed. The average linkage hierarchical clustering was conducted on TOM-based dissimilarity. Clusters were detected by the dynamic hybrid cut method.

Module eigengene was regarded as the dominant component in the principal component analysis of gene expression for the module by eigengene network clustering. In general, the correlations between the modules and mRNAsi were evaluated by Pearson’s correlation. The correlation between genes and mRNAsi was evaluated by gene significance (GS), which was defined as the Pearson’s correlation between gene expression and mRNAsi. The correlation between genes and module was evaluated by module membership (22), which was defined as the Pearson’s correlation between gene expression and module eigengenes. The candidate genes were identified with the threshold: correlation coefficients of GS >0.5, correlation coefficients of MM >0.8.

### Least Absolute Shrinkage and Selection Operator Analysis

We constructed a diagnostic risk score model for HNSCC by candidate genes using the LASSO analysis. The diagnostic risk model was constructed with the following formula: risk score = expression level of Gene_1_ × β1 + expression level of Gene_2_ × β2 +…+ expression level of Gene_n_ × βn. β is the coefficient calculated by LASSO analysis. The receiver operating characteristic (ROC) curves were used to assess the diagnostic value.

The characteristic of LASSO regression is that when the generalized linear model is established, it includes one-dimensional continuous dependent variable, multi-dimensional continuous dependent variable, non-negative number dependent variable, binary discrete dependent variable and multiple discrete dependent variable. In addition, whether the dependent variable is continuous or discrete, LASSO regression can solve it. In general, LASSO regression has a wide range of applications. In addition, LASSO regression can also perform variables screen and reduce the complexity of the model, which refers to controlling the complexity of the model through a series of parameters to avoid overfitting. The complexity of LASSO regression is controlled by λ. The larger the λ, the greater the penalty for the linear model with variables. Thus, a model with fewer variables is finally obtained.

### Analysis of Gene Expression

The expressions of stemness-related genes between normal and tumor samples in RNA-seq data of NJHNCC were evaluated. The validation of stemness-related genes in the TCGA and GEO database was performed. The validation of stemness-related genes in the Oncomine database (http://www.oncomine.org) was performed with threshold: *P =* 0.05, fold change = 2 and gene rank = 10%. The expression of the proteins encoded by the stemness-related genes was analyzed using clinical specimens from the Human Protein Atlas database (https://www.proteinatlas.org) (22).

### Network Analysis of Co-Expressed Genes

Co-expressed genes were obtained from the WGCNA gene module with the threshold: GS >0.5 and MM >0.8. The relationships of co-expressed genes were evaluated in RNA-seq data of NJHNCC using Pearson’s correlation. The protein–protein interactions of co-expressed genes were evaluated in the STRING database (https://string-db.org/). The causal interactions of co-expressed genes were evaluated in the DisNor database (https://disnor.uniroma2.it/). The neighbor genes indicated possible upstream or downstream genes.

### Functional Annotation of Co-Expressed Genes

Co-expressed genes obtained from the WGCNA gene module were used to perform biological function analysis. Gene Ontology (GO) (18) and Kyoto encyclopedia of genes and genomes (KEGG) enrichment analyses were conducted. Biological process (BP), cellular components (CC) and molecular function (MF) were included in GO enrichment analysis. The threshold was *P <*0.05.

### Screening of Potential Compounds

The connectivity map (CMap) database (https://clue.io/cmap) was a comprehensive resource to explore the underlying associations among genes, chemicals, and biological conditions. We utilized the CMap to screen potential compounds that targeting HNSCC stemness genes. We evaluated co-expressed genes and obtained relative compounds.

### Quantitative Real‐Time Polymerase Chain Reaction (qRT‐PCR) Assay

The primary HNSCC tissues and adjacent normal oral epithelial tissues were obtained from 35 HNSCC patients who received radical surgery in the Stomatology Hospital of Nanjing Medical University from January 2019 to June 2020. Frozen tumors and matched normal specimens were confirmed by two independent pathologists. Patients who received chemotherapy, radiotherapy, or any other medical intervention were excluded. Tissue specimens were stored in the −80°C freezer. The study was approved by the institutional review board of Nanjing Medical University.

We extracted the total RNA using TRIzol reagent (Invitrogen, USA) and reagent Kit (TAKARA, Japan) according to the manufacturer’s protocol. The cDNA was reversed by PrimeScript™ RT reagent Kit (TAKARA, Japan). The qRT-PCR was performed on a 7300HT system (ABI, USA) using SYBR Premix Ex Taq II kit (TAKARA, Japan). GAPDH was the reference gene. The relative expression level was calculated by the 2^−ΔΔCt^ method. The sense and antisense primers used are listed below:

GAPDH  5′-GCAAGTTCAACGGCACAG-3′,
      5′-GCCAGTAGACTCCACGACAT-3′
TTK    5′-GTGGAGCAGTACCACTAGAAATG-3′,
      5′-CCCAAGTGAACCGGAAAATGA-3′
KIF14   5′-CCTCACCCACAGTAGCCGA-3′,
      5′-AAGTGCCAATCTACCTACAGGA-3′
KIF18A   5′-TGCTGGGAAGACCCACACTAT-3′,
      5′-GCTGGTGTAAAGTAAGTCCATGA-3′
DLGAP5 5′-AAGTGGGTCGTTATAGACCTGA-3′,
      5′-TGCTCGAACATCACTCTCGTTAT-3′

## Results

### Obtaining and Evaluation of Stemness Index (mRNAsi)

After constructing the predictive model in PCBC stem cell samples and applying it in NJHNCC, we obtained the stemness index (**Supplemental Table 2**). The difference of mRNAsi between 44 tumor and 44 matched normal samples from NJHNCC was evaluated (**Figure 1B**). The mRNAsi of 44 matched normal samples were significantly lower than 44 tumor samples (Paired t-test, *P =* 0.0399). Representative stemness markers (CD44, SOX2) were selected. The good fitness between mRNAsi and stemness markers was observed (**Figures 1C, D**).

### Identification of Candidate Stemness-Related Genes by WGCNA

**Figure 2:**
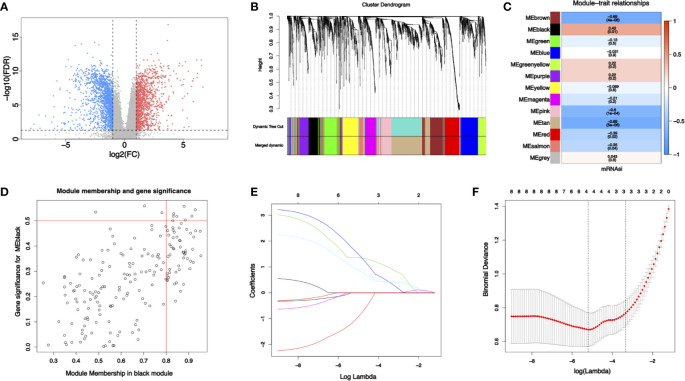
**(A)** Differential gene expression analysis: blue represents downregulated genes and red represents upregulated genes. **(B)** Identification of modules by gene co-expression network in HNSCC. The branches of the cluster dendrogram indicate the modules and the leaves on the cluster dendrogram indicate genes. **(C)** Modules correlated with mRNAsi. The number is the correlated coefficient. The number in parenthesis is the *P* value. **(D)** Scatterplots of module eigengenes. **(E, F)** The least absolute shrinkage and selection operator analysis (LASSO). Coefficient profiles of four stemness-related genes identified to construct risk score model.

Some 2,925 differential expressed genes were obtained by differential gene expression analysis of RNA-seq data from NJHNCC (Wilcoxon test, FDR <0.05), including 1,294 upregulated genes and 1,631 downregulated genes (**Figure 2A** and **Supplemental Table 3**).

To identify biologically significant gene modules related to stemness, WGCNA was performed. Unsupervised hierarchical clustering showed that gene modules were obtained (**Figure 2B**) with soft threshold β = 10 and scale-free R^2^ = 0.9 (**Supplemental Figure 2**). Focusing on cancer stemness, the correlations between modules and mRNAsi were evaluated (**Figure 2C**). The black module turned out to be the most significant module positively correlated with mRNAsi (cor = 0.43, *P =* 0.01). The tan module exhibited a negative correlation with mRNAsi (cor = −0.69, *P <*0.01). Thus, the link between gene module and cancer stemness was established.

With threshold that GS >0.5 and MM >0.8, 8 candidate stemness-related genes in the black module were then identified (**Figure 2D**): cyclin dependent kinase inhibitor 3 (CDKN3), TTK protein kinase (TTK), kinesin family member 14 (KIF14), kinesin family member 18A (KIF18A), DLG associated protein 5 (DLGAP5), kinesin family member (KIF23), cytoskeleton associated protein 2 like (CKAP2L), BUB1 mitotic checkpoint serine/threonine kinase (BUB1). Details of the eight candidate stemness-related genes were shown in **Supplemental Table 4**.

### Identification of Stemness-Related Genes by LASSO and Construction of a Predictive Model for Diagnosis

Some four genes from candidate stemness-related genes were identified using LASSO in RNA-seq data from NJHNCC: TTK, KIF14, KIF18A, and DLGAP5. The coefficients of stemness genes and the minimize λ method screening out four genes were shown (**Figures 2E, F**). The diagnostic risk scores for HNSCC were calculated using the following formula: Risk score = −1.081 × TTK_exp_ + 1.364 × KIF14_exp_ + 1.651 × KIF18A_exp_ + 0.965 × DLGAP5_exp_. The coefficients were obtained from LASSO.

### Evaluation of Stemness-Related Genes’ Expression and Risk Score Model

**Figure 3:**
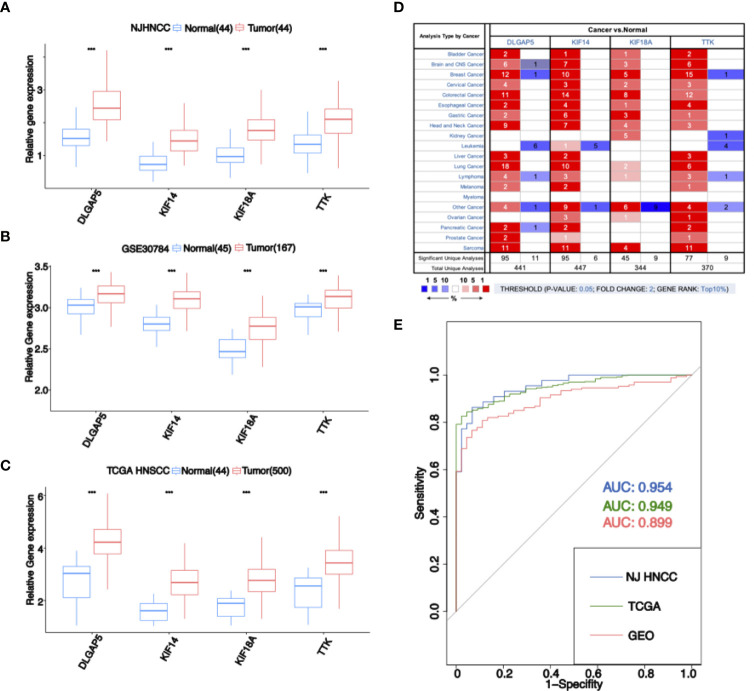
**(A)** Expression of four stemness-related genes in RNA-seq data of NJHNCC. The expression of four stemness-related genes was higher in HNSCC. “***” represents *P <*0.001. **(B)** Expression of four stemness-related genes in GEO dataset. The expression of four stemness-related genes was higher in HNSCC. “***” represents *P <*0.001. **(C)** Expression of four stemness-related genes in TCGA HNSCC dataset. The expression of four stemness-related genes was higher in HNSCC. “***” represents *P <*0.001. **(D)** Expression of four stemness-related genes in Oncomine database. The number in the square is the number of researches meeting the threshold. The color of the square represents the gene rank. Red indicates high expression in tumor samples and blue indicates high expression in normal samples. **(E)** ROC analysis for diagnostic risk score. Blue indicates the result of NJHNCC. Green indicates the result of TCGA. Red indicates the result of GEO.

The expression of stemness-related genes was evaluated in gene expression profiles from NJHNCC (**Figure 3A**), GEO database (**Figure 3B**), TCGA database (**Figure 3C**). Four stemness-related genes were significantly high-expressed in tumor samples (*P <*0.01). In the Oncomine database, the stemness-related genes were upregulated not only in HNSCC but also in other cancers (**Figure 3D**). All of them were in the top 1% of gene rank and each of them had evidence from multiple cancers.

Moreover, we evaluated the risk score model in NJHNCC (AUC = 0.954), TCGA database (AUC = 0.949) and GEO database (AUC = 0.899) (**Figure 3E**).

### Detected Expression of Stemness-Related Genes in HNSCC Tissues

**Figure 4:**
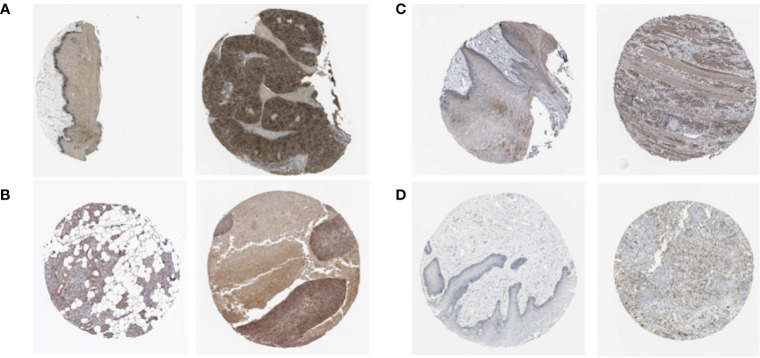
The expression profiles of the proteins encoded by TTK **(A)**, KIF14 **(B)**, KIF18A **(C)** and DLGAP5 **(D)** in normal and HNSCC tissues using clinical specimens from the Human Protein Profiles. The left is normal specimens and the right is specimens from HNSCC patients.

We analyzed the expression of the proteins encoded by the stemness-related genes using clinical specimens from the HPA database. TTK (**Figure 4A**) was moderately positive and KIF14 (**Figure 4B**) was strongly positive in HNSCC metastatic lymph nodes. KIF18A (**Figure 4C**) was moderately positive and DLGAP5 (**Figure 4D**) was strongly positive in HNSCC tissue relative to their expression levels in normal tissue.

### Network Analysis of Co-Expressed Genes

**Figure 5:**
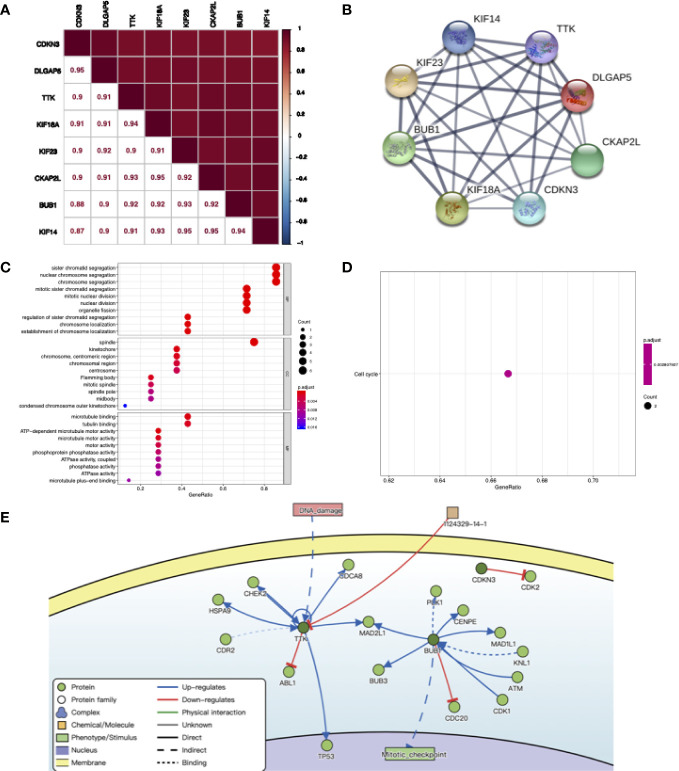
**(A)** The correlation between co-expression genes of stemness-related genes calculating by RNA-seq data of NJHNCC. Pearson correlation coefficient is shown. The depth of square color was determined by the correlation coefficient. **(B)** The relationship of co-expression genes in the STRING database. The thickness of the solid line represents the strength of the correlation. **(C)** Results for GO enrichment analysis. The color of the dot was determined by *P* value. **(D)** Results for KEGG enrichment analysis. The color of the dot was determined by *P* value. **(E)** The causal interactions of co-expression genes in DisNor database. The dark green circles represent co-expression genes. The light green circles represent the neighbor of co-expression genes.

We explored the correlation between eight co-expressed genes by Pearson’s correlation in RNA-seq data of NJHNCC. The result showed that the correlations between the expression of co-expressed genes were significant (*P <*0.05). Each of the stemness genes was highly correlated with other genes (**Figure 5A**). Also, we explored the relationship of co-expressed genes in the STRING database and it showed that they had significant correlations with other genes (**Figure 5B** and **Supplemental Figure 3**).

To investigate the causal interactions of co-expressed genes, we inferred neighbors of them in the DisNor database. The result revealed that PLK1, CENPE and TP53 might be downstream genes. DNA damage might lead to the upregulation of TTK. BUB1 might upregulate mitotic activity (**Figure 5E**).

### Biological Function Analysis of Co-Expressed Genes

To explore the biological function of co-expressed genes, GO and KEGG analyses were performed. The results showed that co-expressed genes were enriched in biological processes including chromosome segregation, nuclear division and organelle fission in GO analysis and cell cycle pathway in KEGG analysis (**Figures 5C, D**).

In addition, genes in the module that negatively related to stemness were also analyzed and the result showed that they were related to extracellular matrix organization (**Supplemental Figures 4A, B**).

### Screening of Potential Compounds for Stemness

**Figure 6:**
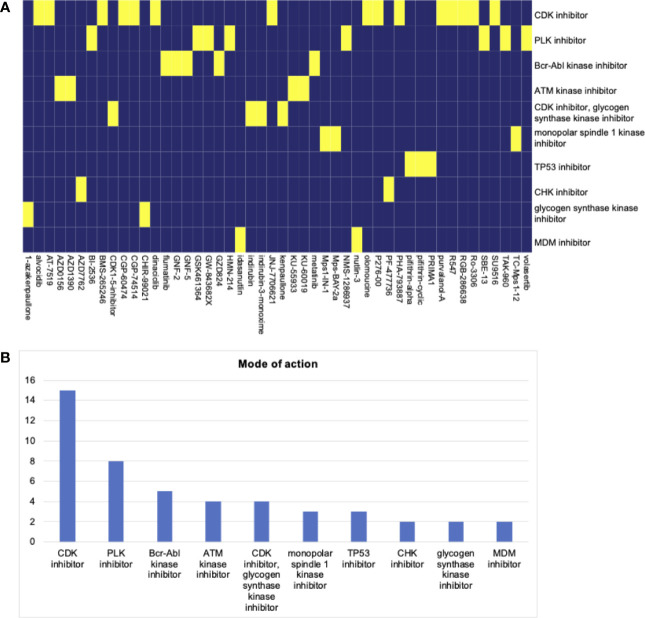
**(A)** The heatmap of compounds from the CMap that share mechanism of action based on co-expression genes. **(B)** The number of genes enriched in each mechanism of action. The top hit is the CDK inhibitor.

We screened potential compounds targeting the stemness of HNSCC in the CMap database. The top compounds were exhibited, along with mechanisms of action (**Figure 6A**). The top mechanism of action was the CDK inhibitor (**Figure 6B**).

### The Expression of Stemness-Related Genes in Tissues

The expressions of stemness-related genes in HNSCC and adjacent normal oral epithelial tissues were evaluated by qRT-PCR (**Supplemental Figure 5**). GAPDH was the reference gene. The expressions of TTK, KIF14, KIF18A and DLGAP5 were upregulated in HNSCC tissues compared to normal tissues (*P <*0.001), which were consistent with our findings.

## Discussion

A recent study has reported that cancer stem cell plays an important role in cancer growth, progression and therapy resistance (23). It indicates that the role of cancer stemness in HNSCC is worth investigating. In this study, we used the machine learning algorithm to learn the gene signature of stem cells and applied it in RNA-seq data of NJHNCC. Then, we identified stemness-related genes of HNSCC and constructed a diagnostic risk score model.

From normal tissue to precancerous lesions and cancer, one essential signature is the process of oncogenic dedifferentiation. The outcome that the stemness of tumor samples was higher than normal samples corroborated this. Some certain mutations contribute to the oncogenesis of dedifferentiated cells rather than mature cells, indicating that cell dedifferentiation status could be one of the oncogenic factors (23). In stemness index analysis, it was associated with stemness marker CD44 and SOX2. The results showed that the stemness index had the ability to distinguish the degree of cancer stemness efficiently.

Next, we obtained candidate stemness-related genes using WGCNA and stemness index. Then we constructed the diagnostic risk score model for HNSCC using LASSO. The predictive model was externally validated and it indicated a robust predictive ability.

With regard to the stemness-related genes, TTK was reported to regulate colon cancer progression *via* PI3K/AKT pathway (24). Kinesins are a family that plays many roles in intracellular transport or cell division. KIF14 and KIF18A were involved in the progression from prometaphase to metaphase stage of mitosis and cytokinesis (25). DLGAP5 was involved in the progress of centrosome-independent mitotic spindle assembly, which is essential for the survival and proliferation of SMARCA4/BRG1 mutant cells. This mechanism was reported in non-small cell lung cancer (26). Among the stemness-related genes, TTK was the cancer driver gene with a significant driver level according to the OncoVar database (27). Though the majority of somatic mutations are harmless, somatic alterations in specific cancer driver genes confer a selective growth advantage for the cells and can lead to cancer (28). It indicates the possibility of investigating cancer stemness in terms of somatic alterations and related cancer driver genes.

With regard to the co-expression network of stemness-related genes, PLK1 promotes EMT and metastasis in gastric carcinoma (29). PLK1 has been identified as a potential target to enhance the therapeutic sensitivity of paclitaxel-resistant prostate cancer (30). CENPE is the largest kinesin protein. The role of CENPE in microtubule kinetochore capture contributes to chromosome congression and alignment (25). Cell division cycle protein was reported to impair ionizing radiation-induced cell apoptosis and promote EMT in nasopharyngeal carcinoma. The expression of CDC6 was upregulated steadily after acute ionizing radiation exposure (31). It indicates that ionizing radiation could lead to oncogenic dedifferentiation, but the mechanism remains unclear.

The signaling pathway that stemness-related genes and co-expression network are most involved in is PI3K/AKT pathway. PI3K/AKT pathway has been reported to regulate EMT (32). Also, the oncogenesis of hepatocellular carcinoma could be activated *via* PI3K/AKT pathway (33). With regard to cancer stemness, research on PI3K/AKT pathway is still lack. Signaling pathways including Hedgehog, Notch, and Wnt pathways have been reported to maintain cancer stemness status (34). Among stemness-related genes and their co-expression network, TTK and PLK1 have been reported to involve in these pathways. In B-cell acute lymphoblastic leukemia, activation of Notch signaling leads to cell-cycle arrest and suppresses PLK1 (35). PLK1 inhibition or depletion enhances the level of cytosolic and nuclear β-catenin in human prostate cancer cells (36). Cell lines harboring activating mutations in the CTNNB1 gene, encoding the Wnt pathway signaling regulator β-catenin, were on average up to five times more sensitive to TTK inhibitors than cell lines wild-type for CTNNB1 (37). EMT has also been involved in the generation of cancer stem cells (38). High expression of the EMT-TFs ZEB1in cancer cells activated the expression of stemness factors SOX2 (39). The ZEB1/miR-200/BMI1 pathway was involved in pancreatic cancer stem cells, which explained how EMT enabled stemness (40). Mesenchymal and stemness traits are known to characterize cancer stemness cell, which is responsible for tumor metastasis and resistance to conventional therapy (41, 42). In stemness-related genes and co-expressed genes we identified, PLK1 and CDC6 have been reported to involve in the progress of EMT (29, 31). The role of other stemness-related genes in EMT is worth investigating.

The potential compounds targeting the HNSCC stemness were screened. Top potential drugs and mechanisms of action were found. The CDK inhibitor was the top hit, following by the PLK inhibitor. To date, High-dose cisplatin, given concurrently with radiotherapy as part of a definitive chemoradiotherapy regimen, is the standard of care, with established survival benefits for patients (43). The compounds targeting CDK and PLK inhibitors may bring novel insights into HNSCC chemotherapy.

In summary, the comprehensive transcriptome profiling of HNSCC using the OCLR algorithm was conducted and stemness-related genes were identified. We also constructed a risk score model for HNSCC. The co-expression network of stemness-related genes was analyzed. CDK and PLK inhibitors play an important role in HNSCC chemotherapy. The application of machine learning in HNSCC transcriptome research brings new perspectives into tumor research. Considering gaps of cell based exploration remained in this study, more experiments need to be conducted to evaluate the effect of stemness-related genes, which will strengthen the elucidation of the diversity and heterogeneity in cancer research. We foresee conducting more researches to further investigate cancer stemness.

## Data Availability Statement

The datasets presented in this study can be found in online repositories. The names of the repository/repositories and accession number(s) can be found below: Figshare Database and accession number NJHNCC-RNAseq-stemsig [14540610] (https://figshare.com/articles/dataset/NJHNCC-RNAseq-stemsig_txt/14540610).

## Ethics Statement

The study was approved by the Institutional Review Board of Nanjing Medical University. The patients/participants provided their written informed consent to participate in this study.

## Author Contributions

All authors listed have made a substantial, direct, and intellectual contribution to the work and approved it for publication.

## Funding

This work was supported in part by the National Natural Science Foundation of China (81672678), Priority Academic Program Development of Jiangsu Higher Education Institutions (PAPD, 2018-87), the Project of Invigorating Health Care through Science, Technology and Education (Jiangsu Provincial Medical Youth Talent QNRC2016852) and sponsored by Qing Lan Project.

## Conflict of Interest

The authors declare that the research was conducted in the absence of any commercial or financial relationships that could be construed as a potential conflict of interest.
