# Supplementary material for: The Identification of Stemness-Related Genes in the Risk of Head and Neck Squamous Cell Carcinoma
Source: Front Oncol. 2021 Jun 11;11:688545. doi: 10.3389/fonc.2021.688545 (PMC8226229; doi:10.3389/fonc.2021.688545)
Supplement: Supplementary file 1 [file DataSheet_1.docx]

**Supplemental Figure 1.** The flowchart of deriving stemness index (mRNAsi) using machine learning method.

**Supplemental Figure 2.** WGCNA(soft threshold β = 10 and scale-free R^2^ = 0.9).

**Supplemental Figure 3.** The relationship of stemness candidate genes in STRING. The number represents the amount of the connections.


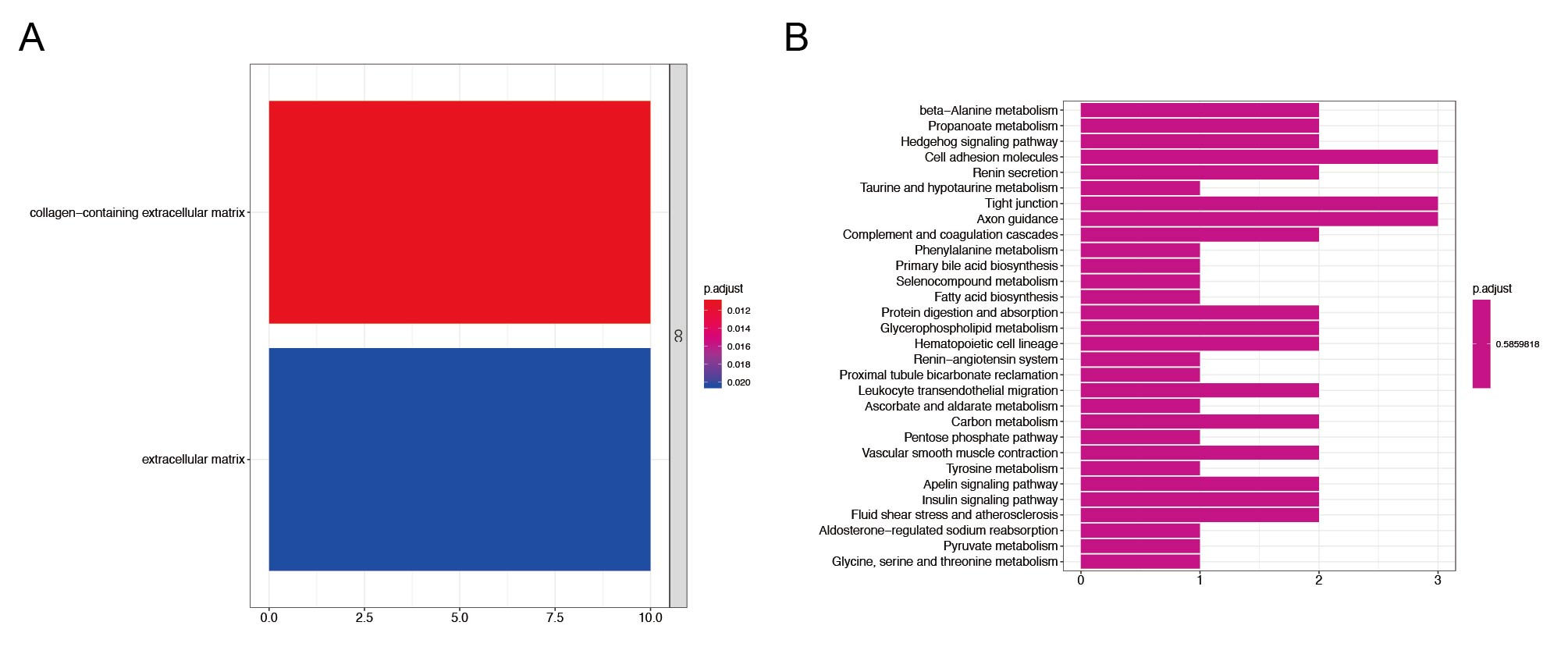


**Supplemental Figure 4.** (A) Results for GO enrichment analysis of tan module. The color of bar was determined by *P* value. (B) Results for KEGG enrichment analysis of tan module. The color of bar was determined by *P* value.

**Supplemental Figure 5.** Validation of stemness-related genes expression using qRT-PCR. Expressions of TTK(A), KIF14(B), KIF18A(C), DLGAP5(D) in adjacent normal oral epithelial tissues and HNSCC tissues (***: *P* <0.001).
