# Supplementary material for: The Identification of Stemness-Related Genes in the Risk of Head and Neck Squamous Cell Carcinoma
Source: Front Oncol. 2021 Jun 11;11:688545. doi: 10.3389/fonc.2021.688545 (PMC8226229; doi:10.3389/fonc.2021.688545)
Supplement: Supplementary file 3 [file DataSheet_3.pdf]

```
####The R code of deriving stemness index(mRNAsi)
####The identification of stemness-related genes in the risk of head and neck squamous cell carcinoma
####Feng et al.

##install packages
install.packages("synapser", repos=c("http://ran.synapse.org", "http://cran.fhcrc.org"))
adeps <- c("gelnet","dplyr","gdata","DT")
for(pkg in deps)    if (!pkg %in% installed.packages()) install.packages(pkg, dependencies = TRUE)

##preparation
library(gelnet)
library(dplyr)
library(biomaRt)
library(synapser)
synLogin('ID','password')

##map ENSEMBL IDs to HUGO
genes2hugo <- function( v, srcType = "ensembl_gene_id" )
```

```
{
   ensembl <- biomaRt::useMart( "ENSEMBL_MART_ENSEMBL", host="www.ensembl.org",
dataset="hsapiens_gene_ensembl" )
   ID <- biomaRt::getBM( attributes=c(srcType, "hgnc_symbol"), filters=srcType, values=v, mart=ensembl )

   j <- which( ID[,2] == "" )
   if( length(j) > 0 ) ID <- ID[-j,]
   stopifnot( all( ID[,1] %in% v ) )
   ID
}

## fnOut - filename of the output signature
## fnGenes - [optional] filename of the list of entrez ID to consider
main.train <- function( fnOut = "pcbc-stemsig.tsv", fnGenes = NULL )
{
   ## Load RNAseq data
   synRNA <- synGet( "syn2701943", downloadLocation = "~/data/PCBC" )
   X <- read.delim( synRNA$path ) %>%
      tibble::column_to_rownames( "tracking_id" ) %>%
```

```
    as.matrix()

## Retrieve metadata
Y <- synMeta %>%
    mutate( UID = gsub("-", ".", UID) ) %>%
    tibble::column_to_rownames( "UID" )

## Retrieve the labels from the metadata
y <- Y[colnames(X),]
names(y) <- colnames(X)

## Fix the missing labels by hand
y["SC11.014BEB.133.5.6.11"] <- "EB"
y["SC12.039ECTO.420.436.92.16"] <- "ECTO"

## Drop the splice form ID from the gene names
v <- strsplit( rownames(X), "\\." ) %>% lapply( "[[", 1 ) %>% unlist()
rownames(X) <- v
```

```
## Map Ensembl IDs to HUGO
V <- genes2hugo( rownames(X) )
X <- X[V[,1],]
rownames(X) <- V[,2]

## Reduce the gene set to the provided list (if applicable)
if( is.null( fnGenes ) == FALSE )
{
   vGenes <- read.delim( fnGenes, header=FALSE ) %>% as.matrix() %>% drop()
   VE <- genes2hugo( vGenes, "entrezgene" )
   X <- X[intersect( rownames(X), VE[,2] ),]
}

## Mean-center the data
m <- apply( X, 1, mean )
X <- X - m

## Identify stem cell samples
j <- which( y == "SC" )
```

```
X.tr <- X[,j]
X.bk <- X[,-j]

## Train a one-class model
mm <- gelnet( t(X.tr), NULL, 0, 1 )

## Store the signature to a file
write.table(mm$w, file = fnOut, sep = "\t", quote = FALSE, col.names = FALSE)

## Perform leave-one-out cross-validation
auc <- c()
for( i in 1:ncol(X.tr) )
{
   ## Train a model on non-left-out data
   X1 <- X.tr[,-i]
   m1 <- gelnet( t(X1), NULL, 0, 1 )

   ## Score the left-out sample against the background
   s.bk <- apply( X.bk, 2, function(z) {cor( m1$w, z, method="sp" )} )
```

```
        s1 <- cor( m1$w, X.tr[,i], method="sp" )

        ## AUC = P( left-out sample is scored above the background )
        auc[i] <- sum( s1 > s.bk ) / length(s.bk)
        cat( "Current AUC: ", auc[i], "\n" )
        cat( "Average AUC: ", mean(auc), "\n" )
    }

    return(auc)
}
main.train( "pcbc-stemsig.tsv")


##load data
p<-read.table('pcbc-stemsig.tsv')
t<-read.table('NJHNCC-RNAseq.txt')
w <-p

## remove the duplicate genes of NJHNCC-RNAseq
```

```
t<- t[!duplicated(t),]
t$V1<-make.names(t$V1,unique = T)
X<-t

## remove the duplication of SLC35E2[optional]
if( length(j) > 1 )
   X <- X[-j[-1],]

##keep common genes
X2<-merge(w,X, by.x ="V1", by.y = "V1")
write.table(X2,"NJHNCC-RNAseq1.txt",sep = "\t",row.names = F)

##load processed data
t<-read.table('NJHNCC-RNAseq1.txt',header = T)

##file rowname conversion
w <-p%>% as.matrix() %>% drop()
rownames(w)<-w[,1]
w<-w[,-1]
```

```
w <-w%>% as.matrix() %>% drop()

##remove the duplicate genes again
t<- t[!duplicated(t),]
t$gene_id<-make.names(t$gene_id,unique = T)
X<-t

##remove the duplication of SLC35E2[optional]
j <- grep( "SLC35E2", X[,1] )
if( length(j) > 1 )
   X <- X[-j[-1],]

## file rowname conversion
rownames(X) <- NULL
X <- X %>% tibble::column_to_rownames( "gene_id" )

##confirm that the genes are the same
stopifnot( all( rownames(X) %in% names(w) ) )
w <- w[ rownames(X) ]
```

```
##file format conversion
w<-as.numeric(w)

##calculate raw stemness index (mRNAsi)
s <- apply( X, 2, function(z) {cor( z, w, method = "sp", use = "complete.obs" )} )

##standardized raw stemness index (mRNAsi)
s <- s - min(s)
s <- s / max(s)

##export stemness index (mRNAsi), only keep values in interval (0,1)
write.table(s, file = "mRNAsi_NJHNCC.txt", sep = "\t", quote = FALSE, col.names = FALSE)
```
